# Sudden Spurt in Pediatric Patients with AKI in Uzbekistan: A Call for International Drug Quality Control and Pharmaceutical Legislation

**DOI:** 10.34067/KID.0000000000000255

**Published:** 2023-10-13

**Authors:** Nodira Murtalibova, Sidharth Kumar Sethi, Rupesh Raina, Bahrom Mamatkulov, Alisher Mustakimov

**Affiliations:** 1 Department of Pediatric Nephrology, National Children Medical Center, Tashkent, Uzbekistan; 2 Pediatric Nephrology, Kidney Institute, Medanta, The Medicity, Gurgaon, India; 3 Pediatric Nephrology, Akron Children's Hospital, Cleveland, Ohio; 4 Tashkent Pediatric Medical Institute, Tashkent, Uzbekistan; 5 Republican Specialized Scientific Research Center of Emergency Medicine, Tashkent, Uzbekistan

**Keywords:** AKI, pediatric nephrology

## Abstract

- These outbreaks serve as a reminder of the difficulties faced in less resourced nations for improvement in regulation and enforcement of quality control procedures in the pharmaceutical industry.
- With the rise in the number of drug manufacturers, monitoring of drug supply in developing countries is extremely important.

An epidemic of severe oligoanuria and altered sensorium has been reported in approximately 120 children from Uzbekistan and adjoining areas of Tajikistan since September 2022. This brief report describes the biochemical and clinical data of 50 patients from two clinical centers from Tashkent, Uzbekistan (Table 1). The age of the affected patients varied from 6 months to 10.5 years. All children had an antecedent history of febrile upper respiratory tract infection, and 36 patients (72%) had taken the same brand of combination medicine of antipyretic and cough suppressant. No other apparent cause could be found in these children, despite a thorough workup for infections, including coronavirus disease–associated multisystemic inflammatory syndrome and H1N1. All patients had severe metabolic acidosis (high anion gap; documented in 15 children; range, 21.7–32.8 mmol/L). Oxalate crystalluria by urine microscopy was documented in 15 of these patients, and high blood ethylene glycol (EG) was documented in 1 patient. Of these, 18 children died and three children are still on hemodialysis currently, and kidney function recovered in the remaining children (*n*=29). These cases represent just the tip of the iceberg because not all children are referred to Tashkent referral centers.

**Table 1:** Clinical profile of pediatric patients admitted from September 2022 to June 2023 (*n*=50)

Clinical & Laboratory Parameters	Data Summary
Age, range	6 mo–10.5 yr
Sex (male)	*n*=28 (56%)
Antecedent febrile upper respiratory tract infection	*n*=50 (100%)
Temporal association (same brand of combination medication taken for fever with cough and cold)	*n*=36 (72%)
Anuria	*n*=48 (96%)
Altered sensorium	*n*=46 (92%)
Metabolic acidosis (pH <7.3)	*n*=50 (100%)
Documented high–anion gap metabolic acidosis	*n*=15 (30%)
Documented oxalate crystalluria	*n*=15 (30%)
High blood EG	*n*=1
Elevated AST, ALT, LDH >10× normal	*n*=47 (94%)
Need for ventilation	*n*=46 (92%)
Need for hemodialysis	*n*=49 (98%)
**Outcomes**	
Ventilator dependence	*n*=4 (8%)
Dialysis dependence	*n*=3 (6%)
Kidney recovery	*n*=29 (58%)
Mortality	*n*=18 (36%)

EG, ethylene glycol; AST, aspartate aminotransferase; ALT, alanine aminotransferase; LDH, lactate dehydrogenase.

Globally, there have been several cases of fatal pediatric AKI linked to the use of cough syrups or acetaminophen and cough syrup combination syrups containing excessive amounts of diethylene glycol (DEG) and EG. Last year, similar cases have been reported in Indonesia (AKI cases in toddlers *n*=325; mortality *n*=174; mortality rate 54.8%) and Gambia (>100 fatalities; mortality rate 84.62% in pediatric patients with AKI) linked to the use of these syrups containing toxic levels of DEG or EG.^1–5^ Similar outbreaks have occurred in the past and can recur in the future and have been typically associated with the contamination of ingestible pharmaceutical products.^1–4^ During the outbreak in Nigeria in 1990, it was found that wholesale distributors substituted DEG for propylene glycol in paracetamol syrups when selling to small pharmaceutical manufacturers to reduce expenses.^6,7^ These outbreaks serve as a reminder of the difficulties faced in less resourced nations for improvement in regulation, enforcement, or strict implementation of quality control procedures in the pharmaceutical industry.^8,9^ Compromises within the pharmaceutical supply chain lead to a proliferation of substandard and falsified (SF) medications. This emphasizes the necessity to evaluate various perspectives through investigations into the structural, political, economic, and ethical elements that shape the quality and utilization of medical products. There is a need for international expansion of formal training on SF medical products to address systemic inadequacies and emphasize the integration of public health education within pharmaceutical training and strengthen the capacity for pharmacists and physicians together to play a pivotal role in safeguarding public health.^8–10^ The intricate interconnection of social and ethical determinants in the issue of SF medicinal products underscores that mere technical interventions are insufficient; instead, it necessitates a comprehensive systemic policy approach enriched and directed by interdisciplinary research across various fields.^8,9^

## Data Availability

All data are included in the manuscript and/or supporting information. The authors have nothing to disclose.
